# An investigation of transmission control measures during the first 50 days of the COVID-19 epidemic in China

**DOI:** 10.1126/science.abb6105

**Published:** 2020-03-31

**Authors:** Huaiyu Tian, Yonghong Liu, Yidan Li, Chieh-Hsi Wu, Bin Chen, Moritz U. G. Kraemer, Bingying Li, Jun Cai, Bo Xu, Qiqi Yang, Ben Wang, Peng Yang, Yujun Cui, Yimeng Song, Pai Zheng, Quanyi Wang, Ottar N. Bjornstad, Ruifu Yang, Bryan T. Grenfell, Oliver G. Pybus, Christopher Dye

**Affiliations:** 1 State Key Laboratory of Remote Sensing Science, College of Global Change and Earth System Science, Beijing Normal University, Beijing, China.; 2 School of Mathematical Sciences, University of Southampton, Southampton, UK.; 3 Department of Land, Air and Water Resources, University of California Davis, Davis, CA, USA.; 4 Department of Zoology, University of Oxford, Oxford, UK.; 5 Harvard Medical School, Harvard University, Boston, MA, USA.; 6 Boston Children’s Hospital, Boston, MA, USA.; 7 Ministry of Education Key Laboratory for Earth System Modeling, Department of Earth System Science, Tsinghua University, Beijing, China.; 8 Beijing Center for Disease Prevention and Control, Beijing, China.; 9 State Key Laboratory of Pathogen and Biosecurity, Beijing Institute of Microbiology and Epidemiology, Beijing, China.; 10 Department of Urban Planning and Design, The University of Hong Kong, Hong Kong.; 11 Department of Occupational and Environmental Health Sciences, School of Public Health, Peking University, China.; 12 Center for Infectious Disease Dynamics, Department of Biology, Pennsylvania State University, University Park, PA, USA.; 13 Department of Entomology, College of Agricultural Sciences, Pennsylvania State University, University Park, PA, USA.; 14 Division of International Epidemiology and Population Studies, Fogarty International Center, National Institutes of Health, Bethesda, MD, USA.; 15 Department of Ecology and Evolutionary Biology, Princeton University, Princeton, NJ, USA.; 16 Oxford Martin School, University of Oxford, Oxford, UK.

## Abstract

Responding to an outbreak of a novel coronavirus [agent of coronavirus disease 2019 (COVID-19)] in December 2019, China banned travel to and from Wuhan city on 23 January 2020 and implemented a national emergency response. We investigated the spread and control of COVID-19 using a data set that included case reports, human movement, and public health interventions. The Wuhan shutdown was associated with the delayed arrival of COVID-19 in other cities by 2.91 days. Cities that implemented control measures preemptively reported fewer cases on average (13.0) in the first week of their outbreaks compared with cities that started control later (20.6). Suspending intracity public transport, closing entertainment venues, and banning public gatherings were associated with reductions in case incidence. The national emergency response appears to have delayed the growth and limited the size of the COVID-19 epidemic in China, averting hundreds of thousands of cases by 19 February (day 50).

## The most effective interventions

By 23 February 2020, China had imposed a national emergency response to restrict travel and impose social distancing measures on its populace in an attempt to inhibit the transmission of severe acute respiratory syndrome–coronavirus 2 (SARS-CoV-2). However, which measures were most effective is uncertain. Tian *et al.* performed a quantitative analysis of the impact of control measures between 31 December 2019 and 19 February 2020, which encompasses the Lunar New Year period when millions of people traveled across China for family visits. Travel restrictions in and out of Wuhan were too late to prevent the spread of the virus to 262 cities within 28 days. However, the epidemic peaked in Hubei province on 4 February 2020, indicating that measures such as closing citywide public transport and entertainment venues and banning public gatherings combined to avert hundreds of thousands of cases of infection. It is unlikely that this decline happened because the supply of susceptible people was exhausted, so relaxing control measures could lead to a resurgence.

*Science*, this issue p. 638

## Teaser

Travel restrictions and the national emergency response delayed the growth and limited the size of the COVID-19 epidemic in China.

On 31 December 2019—less than a month before the 2020 Spring Festival holiday, including the Chinese Lunar New Year—a cluster of pneumonia cases caused by an unknown pathogen was reported in Wuhan, a city of 11 million inhabitants and the largest transport hub in Central China. A novel coronavirus (*1*, *2*) was identified as the etiological agent (*3*, *4*), and human-to-human transmission of the virus [severe acute respiratory syndrome–coronavirus 2 (SARS-CoV-2)] has been since confirmed (*5*, *6*). Further spatial spread of this disease was of great concern in view of the upcoming Spring Festival (*chunyun*), during which there are typically 3 billion travel movements over the 40-day holiday period, which runs from 15 days before the Spring Festival (Chinese Lunar New Year) to 25 days afterward (*7*, *8*).

Because there is currently neither a vaccine nor a specific drug treatment for coronavirus disease 2019 (COVID-19), a range of public health (nonpharmaceutical) interventions has been used to control the epidemic. In an attempt to prevent further dispersal of COVID-19 from its source, all transport was prohibited in and out of Wuhan city from 10:00 a.m. on 23 January 2020, followed by the whole of Hubei Province a day later. In terms of the population covered, this appears to be the largest attempted cordon sanitaire in human history.

On 23 January, China also raised its national public health response to the highest state of emergency: Level 1 of 4 levels of severity in the Chinese Emergency System, defined as an “extremely serious incident” (*9*). As part of the national emergency response, and in addition to the Wuhan city travel ban, suspected and confirmed cases have been isolated, public transport by bus and subway rail suspended, schools and entertainment venues have been closed, public gatherings banned, health checks carried out on migrants (“floating population”), travel prohibited in and out of cities, and information widely disseminated. Despite all of these measures, COVID-19 remains a danger in China. Control measures taken in China potentially hold lessons for other countries around the world.

**Fig. 1:**
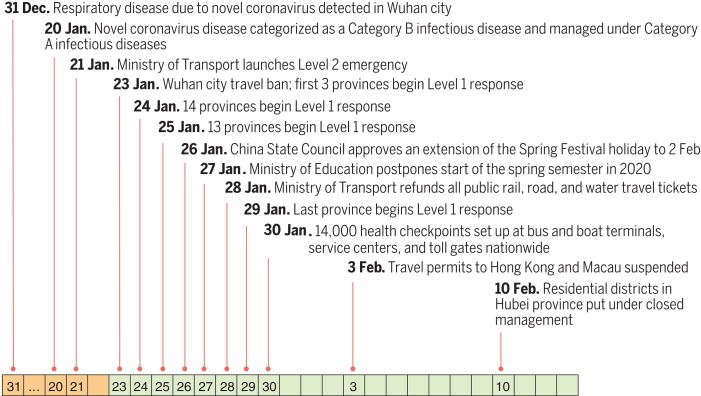
Dates of discovery of the novel coronavirus causing COVID-19 and of the implementation of control measures in China, from 31 December 2019.

Although the spatial spread of infectious diseases has been intensively studied (*10*–*15*), including explicit studies of the role of human movement (*16*, *17*), the effectiveness of travel restrictions and social distancing measures in preventing the spread of infection is uncertain. For COVID-19, SARS-CoV-2 transmission patterns and the impact of interventions are still poorly understood (*6*, *7*). We therefore carried out a quantitative analysis to investigate the role of travel restrictions and transmission control measures during the first 50 days of the COVID-19 epidemic in China, from 31 December 2019 to 19 February 2020 (Fig. 1). This period encompassed the 40 days of the Spring Festival holiday, 15 days before the Chinese Lunar New Year on 25 January and 25 days afterward. The analysis is based on a geocoded repository of data on COVID-19 epidemiology, human movement, and public health (nonpharmaceutical) interventions. These data include the numbers of COVID-19 cases reported each day in each city of China, information on 4.3 million human movements from Wuhan city, and data on the timing and type of transmission control measures implemented across cities of China.

**Fig. 2:**
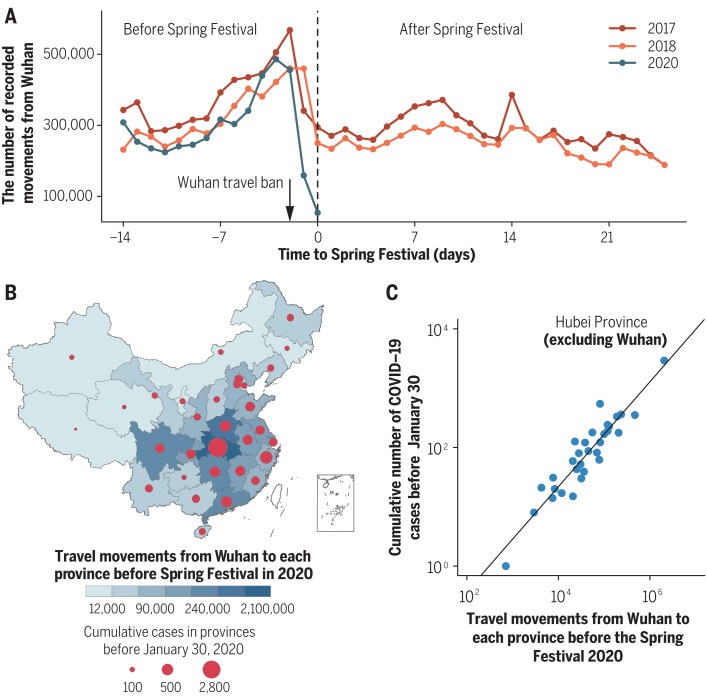
The dispersal of COVID-19 in China 15 days before and 25 days after the Spring Festival (Chinese Lunar New Year). (**A**) Movement outflows from Wuhan city during Spring Festival travel in 2017, 2018, and 2020. The vertical dotted line is the date of the Spring Festival (Chinese Lunar New Year). (**B**) The number of recorded movements from Wuhan city to other provinces during the 15 days before the Spring Festival in 2020. The shading from light to dark represents the number of human movements from Wuhan to each province. The areas of circles represent the cumulative number of cases reported by 30 January 2020, 1 week after the Wuhan travel ban on 23 January. (**C**) Association between the cumulative number of confirmed cases reported before 30 January and the number of movements from Wuhan to other provinces.

We first investigated the role of the Wuhan city travel ban, comparing travel in 2020 with that in previous years and exploring how holiday travel links to the dispersal of infection across China. During Spring Festival travel in 2017 and 2018, there was an average outflow of 5.2 million people from Wuhan city during the 15 days before the Chinese Lunar New Year. In 2020, this travel was interrupted by the Wuhan city shutdown, but 4.3 million people traveled out of the city between 11 January and the implementation of the ban on 23 January (Fig. 2A) (*7*). In 2017 and 2018, travel out of the city during the 25 days after the Chinese Lunar New Year averaged 6.7 million people each year. In 2020, the travel ban prevented almost all of that movement and markedly reduced the number of exportations of COVID-19 from Wuhan (*7*, *8*).

**Fig. 3:**
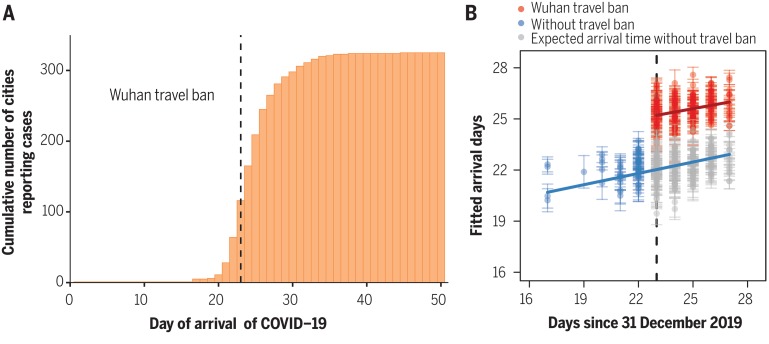
Spatial dispersal of COVID-19 in China. (**A**) Cumulative number of cities reporting cases by 19 February 2020. Arrival days are defined as the time interval (days) from the date of the first case in the first infected city (Wuhan) to the date of the first case in each newly infected city (a total of 324 cities), to characterize the intercity transmission rate of COVID-19. The dashed line indicates the date of the Wuhan travel ban (shutdown). (**B**) Before (blue) and after (red) the intervention by 30 January 2020, 1 week after the Wuhan travel ban (shutdown). The blue line and points show the fitted regression of arrival times up to the shutdown on day 23 (23 January, vertical dashed line). Gray points show the expected arrival times after day 23, without the shutdown. The red line and points show the fitted regression of delayed arrival times after the shutdown on day 23. Each observation (point) represents one city. Error bars give ±2 standard deviations.

The dispersal of COVID-19 from Wuhan was rapid (Fig. 3A). A total of 262 cities reported cases within 28 days. For comparison, the 2009 influenza H1N1 pandemic took 132 days to reach the same number of cities in China (Supplementary materials, materials and methods). The number of cities providing first reports of COVID-19 peaked at 59 per day on 23 January, the date of the Wuhan travel ban.

The total number of cases reported from each province by 30 January, 1 week after the Wuhan shutdown, was strongly associated with the total number of travellers from Wuhan [correlation coefficient (*r*) = 0.98, *P* < 0.01; excluding Hubei, *r* = 0.69, *P* < 0.01] (Fig. 2, B and C). COVID-19 arrived sooner in those cities that had larger populations and had more travellers from Wuhan (Table 1 and table S1). However, the Wuhan travel ban was associated with a delayed arrival time of COVID-19 in other cities by an estimated 2.91 days [95% confidence interval (CI), 2.54 to 3.29 days] on average (Fig. 3B and Table 1).

**Table 1:** Association between the Wuhan travel ban and COVID-19 dispersal to other cities in China. The dependent variable *Y* is the arrival time (days) of the outbreak in each city.

**Covariates**	**Coefficient**	**95% CI**	***P***
Intercept	25.95	(23.43, 28.48)	<0.01
Longitude (degrees)	–0.03	(–0.05, –0.01)	<0.01
Latitude (degrees)	0.03	(0.01, 0.06)	<0.05
log10 (population)	–0.70	(–1.12, –0.28)	<0.01
log10 (total movements)	–0.12	(–0.22, –0.02)	<0.05
Travel ban (days)	2.91	(2.54, 3.29)	<0.01

This delay provided extra time to prepare for the arrival of COVID-19 in more than 130 cities across China but would not have curbed transmission after infection had been exported to new locations from Wuhan. The timing and implementation of emergency control measures in 342 cities across China are shown in Fig. 1 (figs. S2 and S4). School closure, the isolation of suspected and confirmed patients, plus the disclosure of information were implemented in all cities. Public gatherings were banned and entertainment venues closed in 220 cities (64.3%). Intracity public transport was suspended in 136 cities (39.7%), and intercity travel was prohibited by 219 cities (64.0%). All three measures were applied in 136 cities (table S2).

Cities that implemented a Level 1 response (any combination of control measures) (figs. S2 and S4) preemptively, before discovering any COVID-19 cases, reported 33.3% (95% CI, 11.1 to 44.4%) fewer laboratory-confirmed cases during the first week of their outbreaks (13.0 cases; 95% CI, 7.1 to 18.8, *n* = 125 cities) compared with cities that started control later (20.6 cases, 95% CI, 14.5 to 26.8, *n* = 171 cities), with a statistically significant difference between the two groups (Mann-Whitney *U* = 8197, *z* = –3.4, *P* < 0.01). A separate analysis using regression models shows that among specific control measures, cities that suspended intracity public transport and/or closed entertainment venues and banned public gatherings, and did so sooner, had fewer cases during the first week of their outbreaks (Table 2 and table S3). This analysis provided no evidence that the prohibition of travel between cities, which was implemented after and in addition to the Wuhan shutdown on 23 January, reduced the number of cases in other cities across China. These results are robust to the choice of statistical regression model (table S3).

**Table 2:** Associations between the type and timing of transmission control measures and the number of COVID-19 cases reported in city outbreaks the first week. Data were evaluated by means of a generalized linear regression model.

**Covariates**	**Coefficient**	**95% CI**	***P***
(Intercept)	–9.10	(–9.56, –8.64)	<0.01
Arrival time	0.44	(0.43, 0.46)	<0.01
Distance from Wuhan City (log10)	0.61	(0.49, 0.73)	<0.01
Suspension of intra-city public transport			
Implementation	–3.50	(–4.28, –2.73)	<0.01
Timing	0.11	(0.08, 0.14)	<0.01
Closure of entertainment venues			
Implementation	–2.28	(–2.98, –1.57)	<0.01
Timing	0.09	(0.06, 0.11)	<0.01

**Fig. 4:**
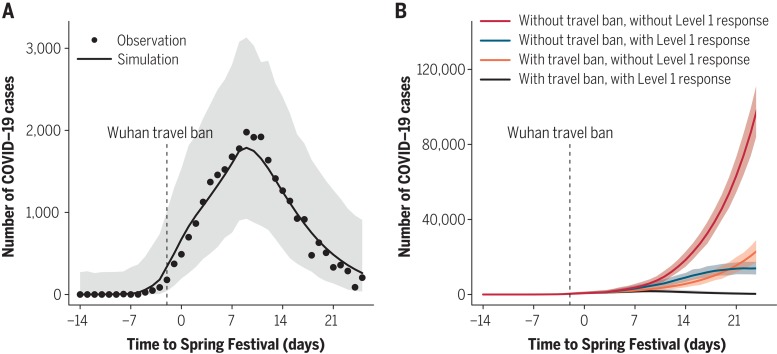
The role of interventions in controlling the COVID-19 outbreak across China. (**A**) Epidemic model (line) fitted to daily reports of confirmed cases (points) summed across 31 provinces. Hubei excludes Wuhan city. (**B**) Expected epidemic trajectories without the Wuhan travel ban (shutdown), and with (blue) or without (red) interventions carried out as part of the Level 1 national emergency response, with the Wuhan travel ban and with (black) or without (orange) the intervention. Vertical dashed lines in (A) and (B) mark the date of the Wuhan travel ban and the start of the emergency response on 23 January. Shaded regions in (A) and (B) mark the 95% prediction envelopes.

The reported daily incidence of confirmed cases peaked in Hubei province (including Wuhan) on 4 February (3156 laboratory-confirmed cases, 5.33 per 100,000 population in Hubei) and in all other provinces on 31 January (875 cases, 0.07 per 100,000 population) (fig. S1). The low level of peak incidence per capita, the early timing of the peak, and the subsequent decline in daily case reports suggest that transmission control measures were associated not only with a delay in the growth of the epidemic but also with a marked reduction in the number of cases. By fitting an epidemic model to the time series of cases reported in each province (fig. S3), we estimate that the (basic) case reproduction number (*R*_0_) was 3.15 before the implementation of the emergency response on 23 January (Table 3). As control was scaled-up from 23 January onward (stage 1), the case reproduction number declined to 0.97, 2.01, and 3.05 (estimated as *C*_1_*R*_0_) in three groups of provinces, depending on the rate of implementation in each group (Table 3 and table S4). Once the implementation of interventions was 95% complete everywhere (stage 2), the case reproduction number had fallen to 0.04 on average (*C*_2_*R*_0_), far below the replacement rate (≪1) and consistent with the rapid decline in incidence (Fig. 4A, Table 3, fig. S5, and table S4).

**Table 3:** Parameter estimates of the SEIR (susceptible-exposed-infectious-recovered) epidemic model. BCI, Bayesian confidence interval; *C*_1_high_, Heilongjiang, Shanghai, Tianjin, Zhejiang, and Hubei (excluding Wuhan); *C*_1_medium_, Anhui, Beijing, Fujian, Guangdong, Guangxi, Guizhou, Hunan, Jilin, Jiangsu, Jiangxi, Inner Mongolia, Shandong, and Tibet; *C*_1_low_, Gansu, Hainan, Hebei, Henan, Liaoning, Ningxia, Qinghai, Shanxi, Shaanxi, Sichuan, Xinjiang, Yunnan, and Chongqing.

**Parameter**	**Definition**	**Mean**	**95% BCI**
ρ	Reporting proportion	0.002	0.001 to 0.003
*R*_0_	Basic reproduction number	3.15	3.04 to 3.26
1/δ	Mean latent period (days)	4.90	4.32 to 5.47
*C*_1_high_	Lower effect of control at the first stage	0.97	0.94 to 0.99
*C*_1_medium_	Medium effect of control at the first stage	0.65	0.58 to 0.72
*C*_1_low_	Higher effect of control at the first stage	0.31	0.24 to 0.38
*C*_2_	Effect of control at the second stage	0.01	0.001 to 0.03
1/γ	Infectious period before isolation (days)	5.19	4.51 to 5.86
*I*_w0_	Minimum number of cases when none detected	1.12	0.91 to 1.32

On the basis of the fit of the model to daily case reports from each province, and on the preceding statistical analyses, we investigated the possible effects of control measures on the trajectory of the epidemic outside Wuhan city (Fig. 4B). Our model suggests that without the Wuhan travel ban or the national emergency response, there would have been 744,000 (±156,000) confirmed COVID-19 cases outside Wuhan by 19 February, day 50 of the epidemic. With the Wuhan travel ban alone, this number would have decreased to 202,000 (±10,000) cases. With the national emergency response alone (without the Wuhan travel ban), the number of cases would have decreased to 199,000 (±8500). Thus, neither of these interventions would, on their own, have reversed the rise in incidence by 19 February (Fig. 4B). But together and interactively, these control measures offer an explanation of why the rise in incidence was halted and reversed, limiting the number of confirmed cases reported to 29,839 (fitted model estimate 28,000 ± 1400 cases), 96% fewer than expected in the absence of interventions.

This analysis shows that transmission control (nonpharmaceutical) measures initiated during the Chinese Spring Festival holiday, including the unprecedented Wuhan city travel ban and the Level 1 national emergency response, were strongly associated with, although not necessarily the cause of, a delay in epidemic growth and a reduction in case numbers during the first 50 days of the COVID-19 epidemic in China.

The number of people who have developed COVID-19 during this epidemic, and therefore the number of people who were protected by control measures, is not known precisely, given that cases were almost certainly underreported. However, in view of the small fraction of people known to have been infected by 19 February (75,532 cases, 5.41 per 100,000 population), it is unlikely that the spread of infection was halted and epidemic growth reversed because the supply of susceptible people had been exhausted. This implies that a large fraction of the Chinese population remains at risk of COVID-19; control measures may need to be reinstated, in some form, if there is a resurgence of transmission. Further investigations are needed to verify that proposition, and population surveys of infection are needed to reveal the true number of people who have been exposed to this novel coronavirus.

We could not investigate the impact of all elements of the national emergency response because many were introduced simultaneously across China. However, this analysis shows that suspending intracity public transport, closing entertainment venues, and banning public gatherings, which were introduced at different times in different places, were associated with the overall containment of the epidemic. However, other factors are likely to have contributed to control, especially the isolation of suspected and confirmed patients and their contact, and it is not yet clear which parts of the national emergency response were most effective. We did not find evidence that prohibiting travel between cities or provinces reduced the numbers of COVID-19 cases outside Wuhan and Hubei, perhaps because such travel bans were implemented as a response to, rather than in anticipation of, the arrival of COVID-19.

This study has drawn inferences not from controlled experiments but from statistical and mathematical analyses of the temporal and spatial variation in case reports, human mobility, and transmission control measures. With that caveat, control measures were strongly associated with the containment of COVID-19, potentially averting hundreds of thousands of cases by 19 February, day 50 of the epidemic. Whether the means and the outcomes of control can be replicated outside China and which of the interventions are most effective are now under intense investigation as the virus continues to spread worldwide.

## Notes

**Correction (8 May 2020):** In the downloadable PDF online, the the correct Fig. 4 image has replaced the repeated Fig. 3 image.

## Supplementary Materials

science.sciencemag.org/content/368/6491/638/suppl/DC1 Materials and Methods Figs. S1 to S7 Tables S1 to S4 References (*19*–*28*)

## Appendix

View/request a protocol for this paper from *Bio-protocol*.
